# Complete mitochondrial genome of *Bugula neritina *(Bryozoa, Gymnolaemata, Cheilostomata): phylogenetic position of Bryozoa and phylogeny of lophophorates within the Lophotrochozoa

**DOI:** 10.1186/1471-2164-10-167

**Published:** 2009-04-21

**Authors:** Kuem Hee Jang, Ui Wook Hwang

**Affiliations:** 1 Department of Biology, Graduate School & Department of Biology, Teachers College, Kyungpook National University, Daegu 702-701, Korea; 2 Institute for Phylogenomics and Evolution, Kyungpook National University, Daegu 702-701, Korea

## Abstract

**Background:**

The phylogenetic position of Bryozoa is one of the most controversial issues in metazoan phylogeny. In an attempt to address this issue, the first bryozoan mitochondrial genome from *Flustrellidra hispida *(Gymnolaemata, Ctenostomata) was recently sequenced and characterized. Unfortunately, it has extensive gene translocation and extremely reduced size. In addition, the phylogenies obtained from the result were conflicting, so they failed to assign a reliable phylogenetic position to Bryozoa or to clarify lophophorate phylogeny. Thus, it is necessary to characterize further mitochondrial genomes from slowly-evolving bryozoans to obtain a more credible lophophorate phylogeny.

**Results:**

The complete mitochondrial genome (15,433 bp) of *Bugula neritina *(Bryozoa, Gymnolaemata, Cheilostomata), one of the most widely distributed cheliostome bryozoans, is sequenced. This second bryozoan mitochondrial genome contains the set of 37 components generally observed in other metazoans, differing from that of *F. hispida *(Bryozoa, Gymnolaemata, Ctenostomata), which has only 36 components with loss of tRNA^ser(ucn) ^genes. The *B. neritina *mitochondrial genome possesses 27 multiple noncoding regions. The gene order is more similar to those of the two remaining lophophorate phyla (Brachiopoda and Phoronida) and a chiton *Katharina tunicate *than to that of *F. hispida*. Phylogenetic analyses based on the nucleotide sequences or amino acid residues of 12 protein-coding genes showed consistently that, within the Lophotrochozoa, the monophyly of the bryozoan class Gymnolaemata (*B. neritina *and *F. hispida*) was strongly supported and the bryozoan clade was grouped with brachiopods. Echiura appeared as a subtaxon of Annelida, and Entoprocta as a sister taxon of Phoronida. The clade of Bryozoa + Brachiopoda was clustered with either the clade of Annelida-Echiura or that of Phoronida + Entoprocta.

**Conclusion:**

This study presents the complete mitochondrial genome of a cheliostome bryozoan, *B. neritina*. The phylogenetic analyses suggest a close relationship between Bryozoa and Brachiopoda within the Lophotrochozoa. However, the sister group of Bryozoa + Brachiopoda is still ambiguous, although it has some attractions with Annelida-Echiura or Phoronida + Entoprocta. If the latter is a true phylogeny, lophophorate monophyly including Entoprocta is supported. Consequently, the present results imply that Brachiozoa (= Brachiopoda + Phoronida) and the recently-resurrected Bryozoa concept comprising Ectoprocta and Entoprocta may be refuted.

## Background

Bryozoans (ectoprocts), also known as "moss animals", are aquatic organisms that mostly live in colonies of interconnected individuals. They usually encrust rocky surfaces, shells or algae. They are an ecologically important group, with the marine species forming a dominant component of benthic subtidal marine communities. This group is also economically important because it is a major component of both marine and freshwater biofouling, and evolutionarily important as a long-living phylum with a good fossil record [1]. The phylum is currently reported to contain 4000 extant species. However, it is likely that more than twice that number are currently in existence [2,3], with new taxa being described annually.

Together with the Brachiopoda and Phoronida, Bryozoa have been classified as "Lophophorata" because they possess a similar suspension feeding apparatus, the lophophore, which is a horseshoe-shaped structure that surrounds the mouth and has ciliated tentacles [4-8]. However, lophophorate phylogeny remains one of the most controversial issues in metazoan animal phylogeny because they display an amalgam of deuterostome and protostome features. The "Lophophorata" have been classified as deuterostomes on the basis of morphological and larval features [9-13]. On the other hand, molecular phylogenetic analyses suggest that the lophophorates have some affinities with mollusks and annelids within the protostomes [14-21].

Lophophorate phylogenies that have been reconstructed with mitochondrial protein-coding genes and nuclear ribosomal DNAs have failed to resolve the detailed relationships among the lophophorates and other related metazoan phyla [15,17,22-24]. Most studies of complete mitochondrial genomes have focused on chordate and arthropod phylogenies because only a few mitochondrial genomes from lophotrochozoan phyla have been determined to date. So far, complete lophotrochozoan mitochondrial genome sequences have been published for 94 species from 12 phyla, including 45 mollusks, 8 annelids, 3 brachiopods, 1 bryozoan, 1 phoronid (nearly complete), 2 entoprocts, 28 platyheminths, 1 nemertean (nearly complete), 1 rotifer, 2 chaetognaths, 1 acanthocephalan and 1 echiuran. If the mollusk data are excluded, only 49 mitochondrial genomes have been sequenced from the huge protostome group (the Lophotrochozoa) so far.

Complete mitochondrial genomes have been characterized from a variety of metazoan phyla so that nucleotide, amino acid and gene order data can be used to resolve their phylogenetic relationships. Mitochondrial genomes are generally conserved in terms of gene components (usually 13 protein-coding genes, 2 ribosomal RNA genes and 22 transfer RNA genes) [25], and a number of studies have taken advantage of the various levels of phylogenetic information offered by mitochondrial genomes to solve systematic and evolutionary questions over a broad taxonomic range [26,27].

Mitochondrial protein-coding genes have recently been used to resolve the phylogenetic relationships of lophophorates [28]. The results show that the phylum Brachiopoda (an articulate brachiopod, *Terebratulina retusa*) belongs to the lophotrochozoan protostomes and that Brachiopoda have a close relationship with Molluska and Annelida within the monophyletic clade, Lophotrochozoa. The second lophophorate phylum, Phoronida (*Phoronis architecta*), has also been placed within the Lophotrochozoa. *Phoronis *has the almost same gene arrangement as the chiton, *Katharina tunicata *(Molluska, Polyplacophora) [29]. Phylogenies based on most of the molecular data strongly suggest that two lophophorate phyla, Brachiopoda and Phoronida, are closely related to each other (called Phoronizoa or Brachiozoa), and they appear to be sister groups of mollusks and annelids within the Lophotrochozoa [11,30].

In an attempt to address the phylogenetic position of bryozoans in metazoan phylogeny, the first mitochondrial genome from a ctenostome bryozoan, *Flustrellidra hispida *(Flustrellidridae), was recently sequenced and characterized. However, *F. hispida *exhibits a number of peculiar features, such as extensive translocation of gene components including protein-coding and tRNA genes, and extremely reduced size. Phylogenetic trees inferred from the nucleotide and amino acid sequences of its protein-coding genes were mutually conflicting, so the phylogenetic position of *F. hispida *was not assigned. Thus, it is necessary to sequence additional mitochondrial genomes from more representative and widely-distributed bryozoans in order to address the issue of the phylogenetic position of bryozoans on the basis of mitochondrial genome information.

In this paper, to address whether or not lophophorates are a monophyletic group and to examine the exact phylogenetic position of Bryozoa, we describe the complete mitochondrial genome sequence of *Bugula neritina *(Bryozoa, Gymnolaemata, Cheilostomata), one of the most widely-distributed cheliostome bryozoans. The result is compared with the *F. hispida *sequence. We also explore the following: the monophyly of the class Gymnolaemata, the phylogenetic implication of the gene orders in lophophorate mitochondrial genomes, the secondary structures of extremely multiplied noncoding regions, etc.

## Results and discussion

### Genome organization

**Figure 1:**
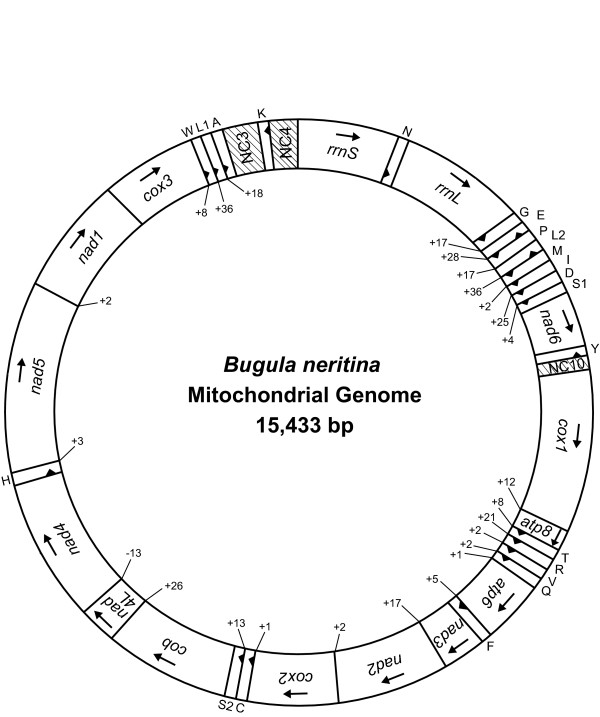
**A circular map of the complete mitochondrial genome of a bryozoan *Bugula neritina *(GenBank accession number **AY690838). Protein and rRNA genes are abbreviated as follows: *atp6 *and *atp8 *(genes for ATPase subunits 6 and 8), *cox1-cox3 *(genes for cytochrome C oxidase subunits I-III), *cob *(gene for apocytochrome b), *nad1-nad6 *and *nad4L *(genes for NADH dehydrogenase subunits 1–6 and 4L), and *rrnS *and *rrnL *(genes for 12S and 16S rRNAs). All 22 tRNA genes are located among protein- and/or tRNA-coding genes. The tRNA genes are named using single-letter amino acid abbreviations, with the exception of those coding for leucine and serine, which are named *L1 *for the tRNA^Leu(CUN) ^(anticodon TAG) gene, *L2 *for the tRNA^Leu(UUR) ^(anticodon TAA) gene, *S1 *for the tRNA^Ser(AGN) ^(anticodon GCT) gene and *S2 *for the tRNA^Ser(UCN) ^(anticodon TGA) gene. The arrows indicate the orientations of the gene components. The three slashed regions corresponding to NC3, NC4 and NC10 may be related to the mode of regulation of mitochondrial replication and transcription.

The mitochondrial genome sequence of *Bugula neritina *is 15,433 bp long and consists of 13 protein-coding genes (*cox1-3*, *nad1-nad6*, *nad4L*, *atp6*, *atp8 *and *cob*), two rRNA genes for the small and large subunits (*rrnS *and *rrnL*), and 22 tRNA genes, as is typical of the animal mitochondrial genomes published so far (Fig. 1). The A+T content of the entire mitochondrial genome of *B. neritina *is 70.0%. Interestingly, we found 27 multiplied noncoding regions (NC1-27). All the protein-coding and rRNA genes and 17 of the tRNA genes are transcribed in the same strand in *B. neritina*; the other five tRNAs are [*trnL*(*cun*), *trnA*, *trnE*, *trnY *and *trnV*], (Fig. 1). The first bryozoan mitochondrial genome reported from *F. hispida *[31] has only 36 gene components because *trnS(ucn) *is absent, it is relatively short (13,026 bp), and the A+T content is lower (59.4%). In contrast, *B. neritina *has features that are more typical of metazoan mitochondrial genomes in general in terms of the number of gene components, whole genome size and A+T content.

### Extreme multiplication of noncoding region

**Figure 2:**
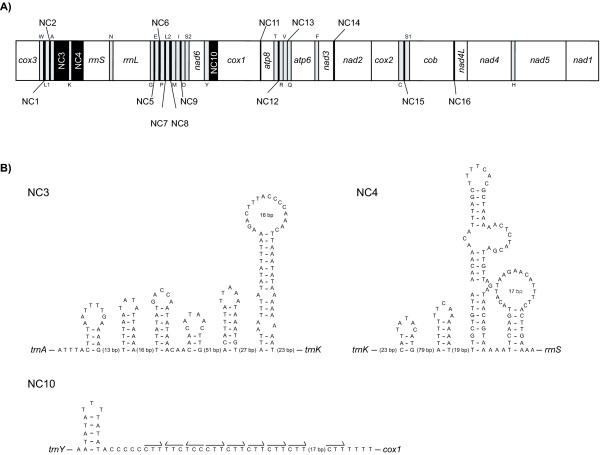
**Multiple noncoding regions of the mitochondrial genome of a bryozoan, *Bugula neritina*, putative secondary structures of NC3, NC4 and NC10, and "CTT" repeat motif observed in NC10**. A) Fifteen (NC1-NC16) larger than 10 bp of the 27 multiple noncoding regions of the *Bugula neritina *mitochondrial genome (*black boxes*). The circular genome is linearized. Genes encoded on the opposite strand are shown in *gray boxes*. NC3, NC4 and NC10 may be related to the mode of regulation of mitochondrial replication and transcription. B) Plausible helix structures predicted from NC3, NC4 and NC10, and 9 "CTT" repeats observed in NC10. The secondary structures and repeats may play important roles in the regulation of mitochondrial replication and transcription. Arabic numbers inside the encircled loop regions of each helix and in parentheses between helices indicate the number of nucleotides in each region.

Strikingly, the *B. neritina *mitochondrial genome contains 27 multiplied noncoding regions: 16 noncoding regions (NC1-NC16) larger than 10 bp (Table 1 and Fig. 2A) and 11 smaller (Table 1). The total length of the 16 noncoding regions larger than 10 bp is 864 bp. Three of them – NC3 (271 bp) between *trnA *and *trnK*, NC4 (246 bp) between *trnK *and *rrnS *and NC10 (68 bp) between *trnY *and *cox1 *– could be candidate origins of replication. *trnK*, one of the five tRNA genes transcribed on the light strand, is located between NC3 and NC4. The placement of *trnK *between these two possible control regions is likely to have occurred very recently and independently only in the specific evolutionary lineage of *B. neritina*, since it has never been found in any other metazoan. The remaining 13 noncoding regions (NC1-NC2, NC5-NC9, NC11-NC16) total 279 bp in length and are dispersed throughout the whole genome, ranging from 12 to 36 bp in size (Table 1 and Fig. 2A). In addition, 11 small intergenic gaps (< 10 bp) were identified between some gene components (Table 1).

**Table 1:** The mitochondrial genome profile of *Bugula neritina*

	**Positions**				**Codons**		
					
**Features**	**From**	**To**	**Strands**	**Sizes** **(bp)**	**Start**	**Stop**	**Intergenic nucleotides**^a^
*cox3*	1	822	+	822	ATG	TAA	8
*trnW*	831	897	+	67			34
*trnL1*	932	985	-	54			18
*trnA*	1004	1065	-	62			0
*NC3*	1066	1336		271			0
*trnK*	1337	1405	+	69			0
*NC4*	1406	1651		246			0
*rrnS*	1652	2491		840			0
*trnN*	2492	2555	+	64			0
*rrnL*	2556	3882		1327			0
*trnG*	3883	3947	+	65			17
*trnE*	3965	4025	-	61			28
*trnP*	4054	4121	+	68			17
*trnL2*	4139	4199	+	61			36
*trnM*	4236	4298	+	63			2
*trnI*	4301	4367	+	67			25
*trnD*	4393	4459	+	67			4
*trnS1*	4464	4523	+	60			0
*nad6*	4524	4993	+	470	ATG	TA*	0
*trnY*	4994	5038	-	45			0
*NC10*	5039	5106		68			0
*cox1*	5107	6642	+	1536	ATA	TAA	12
*atp8*	6655	6780	+	126	ATG	TAA	8
*trnT*	6789	6854	+	66			21
*trnR*	6876	6941	+	66			2
*trnV*	6944	6996	-	53			15
*trnQ*	7012	7072	+	61			1
*atp6*	7074	7763	+	690	ATG	TAA	5
*trnF*	7769	7834	+	66			0
*nad3*	7835	8188	+	354	ATG	TAA	17
*nad2*	8206	9141	+	936	ATG	TAA	2
*cox2*	9144	9815	+	672	ATG	TAA	1
*trnC*	9817	9878	+	62			13
*trnS2*	9892	9950	+	59			0
*cob*	9951	11057	+	1107	ATG	TAA	26
*nad4L*	11084	11389	+	306	ATT	TAA	-13
*Nad4*	11377	12733	+	1357	ATT	T*	0
*trnH*	12734	12797	+	64			3
*nad5*	12801	14495	+	1695	ATG	TAA	2
*nad1*	14498	15433	+	936	ATG	TAA	0

^a^: Gap nucleotides (positive value) or overlapped nucleotides (negative value) between adjacent genes.

*: Incomplete termination codon, which is probably extended by post-transcriptional adenylation.

Most metazoan mitochondrial genomes reported so far possess only a single major noncoding region, which is thought to be involved in the regulation of transcription and the control of DNA replication [32,33]. In general, possible control regions possess characteristic features such as high A+T contents, hairpin-loop structures, repeat motifs, etc. [25,34]. In *B. neritina*, there are three possible control regions (NC3, NC4 and NC10). Their A+T contents are 78.6% in NC3, 78.1% in NC4 and 79.4% in NC10, all of which are much higher than the 70.0% of the mitochondrial genome as a whole. In NC3, NC4 and NC10, we found some hairpin-loop structures that might be related to the mode of regulation of replication and transcription (Fig. 2B). NC3 and NC4 possess no characteristic repeat motifs but have extensive poly "A" and poly "C" tracts (136 "A" and 12 "C" in NC3 and 122 "A" and 36 "C" in NC4), as often observed in mitochondrial control regions in other metazoans [25,34]. Intriguingly, NC10 (12 A, 15 C, 2 G and 37 T) includes at least nine "CTT" repeats with a short helix consisting of a 5-base-pair stem and a 3-nt loop (Fig. 2B). Despite its short length (68 bp), the existence of "CTT" repeats and a hairpin-loop may suggest that NC10 is important in regulating mitochondrial replication and transcription. In addition to these, NC1 between *trnW *and *trnL*(*cun*) has a helix with a 5-bp stem [additional file 1].

Such multiple noncoding regions are rare in metazoan mitochondrial genomes. The other bryozoan sequenced, *F. hispida*, has 17 noncoding regions, ranging in size from 1 to 195 bp (506 bp in total). Among these, two possible control regions were observed between *trnC *and *trnG *(195 bp) and between *cox2 *and *trnD *(114 bp), which are separated by *cox2*-*trnG *[31]. The mollusk *Loligo bleekeri *(Cephalopoda; [35]) has 19 noncoding regions longer than 10 bp. Three of these 19 are 515 bp, 507 bp and 509 bp long, and their sequences are nearly identical, suggesting that all three originated from a single, large, ancestral noncoding region. In *Lampsilis ornata *(Bivalvia; [36]), 28 noncoding regions were found, ranging from 2 to 282 bp in size. Of these, only one large noncoding region (136 bp long) has an increased A+T content (76.8%), so it is a possible control region. Since no such extreme multiplication of noncoding regions has been observed in any other bivalve or cephalopod mollusk including *Katharina tunicata*, it is likely that the extreme multiplication of noncoding regions is a homoplasious characteristic, occurring independently in the lineages of *L. bleekeri*, *L. ornata *and *B. neritina*.

### Comparative analysis of gene arrangements

Unlike other metazoan mitochondrial genomes in which genes are encoded on both strands, all the protein-coding and rRNA genes and 17 of the tRNA genes – the exceptions being the five tRNA genes *trnL*(*cun*), *trnA*, *trnE*, *trnY *and *trnV *– are transcribed from the same strand in *B. neritina *(Fig. 1 and Table 1). In *F. hispida*, one protein-coding gene (*cox2*), one ribosomal RNA gene (*rrnL*) and four tRNA genes (*trnG*, *trnC*, *trnL(uur)*, *trnV *and *trnV*) are reversed. Such a single-strand-dependent transcription tendency has been reported for 137 among the 1428 metazoan species in 23 phyla for which complete or nearly complete mitochondrial genome sequences have been determined to date (Dec. 17, 2008). Except for six tunicates (Deuterostomia, Urochordata), all the remaining 131 cases were from protostomes or primitive metazoan groups: 83 protostomes including 62 lophotrochozoans and 17 nematodes, and 48 primitive metazoans including 29 cnidarians and 19 poriferans, the most primitive metazoan groups (Table 2). The single-strand dependence of transcription might be a plesiomorphic, ancestral characteristic because such a tendency appears in 48 out of 59 primitive metazoans (81.4%) such as Cnidaria and Porifera (Table 2).

**Table 2:** List of metazoan mitochondrial genomes showing single-strand dependent transcription tendency for protein-coding and ribosomal RNA genes

**Classifications**	**Complete mitochondrial genomes**^1)^	**Single-strand dependency**^2)^	**Species names**
**Primitive metazoans**			
Cnidaria	34	29	*Metridium senile *etc.
Porifera	21	19	*Tethya actinia *etc.
Others	4	0	
**Deuterostomia**			
Urochordata	6	6	*Ciona intestinalis *etc.
Others	1031	0	
**Protostomia**			
**Lophotrochozoa**			
**Bryozoa**	**2**	**1**	***Bugula neritina***
			***Flustrellidra hispidia***
**Brachiopoda**	**3**	**3**	***Terebratulina retusa***
			***Laqueus rubellus***
			***Terebratalia transversa***
**Phoronida**	**1**	**0**	***Phoronis psamophila***
Entoprocta	2	0	
Annelida	8	8	*Platynereis dumerilii *etc.
Molluska	45	18	*Mytilus edulis *etc.
Platyhelminthes	28	28	*Schistosoma japonicum *etc.
Echiura	1	1	*Urechis caupo*
Chaetognatha	2	0	
Nemertea	1	1	*Cephalothrix rufifrons*
Acanthocephala	1	1	*Leptorhychoides thecatus*
Rotifera	1	1	*Brachionus plicatilis*
**Ecdysozoa**			
Nematoda	27	17	*Caenorhabditis elegans *etc
Arthropoda	207	4	*Tigriopus califormicus *etc.
Others	3	0	

**Total**	**1428**	**137**	

^1) ^The number of mitochondrial genomes completely sequenced to date

^2^) The number of mitochondrial genomes showing single-strand dependent transcription tendency

**Figure 3:**
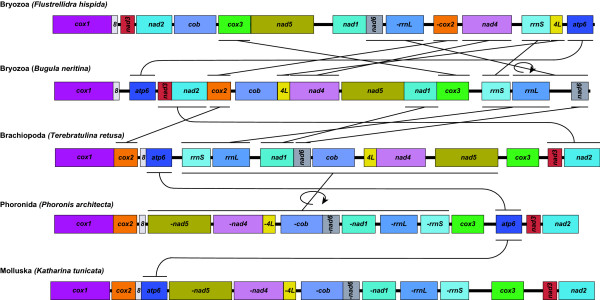
**Comparison of arrangement of the mitochondrial protein-coding and ribosomal RNA genes for 2 bryozoans, 1 brachiopod, 1 phoronid and 1 polyplacophoran**. Protein-coding and ribosomal RNA genes are designated by their abbreviations as shown in Fig. 1. Each gene map commences from *cox3 *and is oriented so that the gene is transcribed from left to right. The rearrangements that are needed to inter-convert the pair of maps are shown, disregarding tRNA genes in which shared gene arrangements are indicated. A circular arrow indicates inversion of a single gene or a block consisting of more than two genes. Dramatic differences were found in tRNA gene positions, but they are not depicted because they are highly complex.

The arrangements of the protein-coding and rRNA genes were compared among two bryozoans (*B. neritina *and *F. hispidia*), a brachiopod (*T. retusa*), a phoronid (*P. architecta*) and a polyplachophoran (*K. tunicata*) (Fig. 3). The overall gene arrangement in *B. neritina *was quite different from those in other metazoans published so far. Compared to the *F. hispida *sequence, *B. neritina *needed 6 local translocations and 1 inversion to have the same gene order. On the other hand, only 5 translocations from a brachiopod, *T. retusa*, and 6 translocations with 1 inversion from a phoronid, *P. architecta*, would produce the gene arrangement of *B. neritina*; therefore, the gene arrangement in *T. retusa *is most similar to that of *B. neritina*. The *B. neritina *gene arrangement could be obtained from that of *T. retusa *by only five translocation events (*rrnS/rrnL*, *nad3/nad2*, *cox2*, *nad1 *and *nad6*) with no inversions. The phoronid gene arrangement was identical to that of *Katharina *with only one exception, a difference in the position of *atp6*.

### Nucleotide composition and codon usage

As shown in Table 3, the overall A+T content of the *B. neritina *mitochondrial genome is 70.0% (+ strand: A = 37.7%; C = 17.6%; G = 12.4%; T = 32.3%), which is typical of the base compositions of metazoan mitochondrial genomes. However, it is unusual in comparison to those of other bryozoans and brachiopods; it is much higher than those of *F. hispida *(59.4%) and of three brachiopods, *T. retusa *(57.2%), *T. transversa *(59.1%) and *L. rubellus *(58.3%).

**Table 3:** Nucleotide compositions and AT- and CG-skews of the mitochondrial protein-coding and ribosomal RNA genes and the entire *Bugula neritina *genome

	**Proportion of nucleotides**						
				
**Gene**	**A**	**C**	**G**	**T**	**AT%**	**AT skew**	**CG skew**
atp6 (+)	0.316	0.190	0.142	0.352			
atp8 (+)	0.365	0.175	0.056	0.405	77.0	-0.052	0.513
cox1 (+)	0.297	0.182	0.174	0.348	64.5	-0.079	0.020
cox2 (+)	0.360	0.192	0.150	0.298	65.8	0.094	0.123
cox3 (+)	0.349	0.190	0.153	0.308	65.7	0.062	0.108
cob (+)	0.343	0.189	0.127	0.341	68.4	0.003	0.196
nad1 (+)	0.364	0.203	0.124	0.309	67.3	0.082	0.242
nad2 (+)	0.372	0.183	0.103	0.343	71.5	0.041	0.277
nad3 (+)	0.322	0.169	0.136	0.373	69.5	-0.073	0.108
nad4 (+)	0.384	0.178	0.108	0.329	71.3	0.077	0.247
nad4L (+)	0.386	0.141	0.098	0.376	76.2	0.013	0.176
nad5 (+)	0.395	0.190	0.106	0.310	70.5	0.121	0.281
nad6 (+)	0.349	0.160	0.102	0.389	73.8	-0.054	0.221
rrnL(+)	0.433	0.145	0.136	0.287	72.0	0.203	0.029
rrnS (+)	0.420	0.164	0.145	0.271	69.1	0.216	0.061
							
Entire genome	0.377	0.176	0.124	0.323	70.0	0.078	0.173

AT skew = (A%-T%)/(A%+T%); CG skew = (C%-G%)/(C%+G%)

Table 3 shows the AT- and CG-skews of each of the 13 protein-coding and 2 ribosomal RNA genes and of the whole genome (total) in *B. neritina *mitochondria. The results show no marked bias in nucleotide composition. The AT-skew is positive for 11 genes and negative for five on the (+) strand. The CG-skew for all 15 genes on the (+) strand is positive. This means that the *B. neritina *mitochondrial genome has no biased nucleotide composition. As shown in [additional files 2 and 3], the other bryozoan, *F. hispida*, has no biased nucleotide composition either. In contrast, the AT-skews of 12 genes in *T. transversa *and *L. rubellus *and the CG-skews of nine genes in all three brachiopods seem clearly biased.

The codon usage pattern of the *B. neritina *mitochondrial protein-coding genes is shown in Table 4. There is a clear preference for A+T-rich codons; the five most frequently used codons are UUA (300 times) for leucine, AUA (281) for methionine, AUU (237) for isoleucine, UUU (178) for phenylalanine and AAA (144) for lysine. Compared to other lophotrochozoans, the *B. neritina *mitochondrial genome showed a strong bias to A+T codons with dramatically lower G+C content. The anticodon nucleotides in *B. neritina *were completely identical to those of the brachiopod *Laqueus rubellus *[37] and the annelid *Lumbricus terrestris *[38] except for *trnL(cun) *and *trnY*. However, two anticodons – UUU in *trnK *and UCU in *trnS(agn) *– in *B. neritina *were different from those used in most other metazoans. The tRNA anticodon corresponding to the codon AGN for serine is UCU, as in nematode mitochondrial genomes, but in most other metazoan mitochondrial genomes such as those of platyhelminthes, mollusks, *Drosophila *and echinoderms, the serine tRNA anticodon is GCU rather than UCU [25,38].

**Table 4:** Codon usage pattern of 13 mitochondrial protein-coding genes in *Bugula neritina*

**Amino acid**	**Codon**	**N**^a^	**Amino acid**	**Codon**	**N**^a^	**Amino acid**	**Codon**	**N**^a^	**Amino acid**	**Codon**	**N**^a^
Phe	UUU	178	Ser	UCU	69	Tyr	UAU	58	Cys	UGU	17
(GAA)	UUC	66	(UGA)	UCC	40	(AUA)	UAC	63	(GCA)	UGC	14
											
Leu	UUA	300		UCA	77	Ter	UAA	11	Trp	UGA	72
(UAA)	UUG	36		UCG	6		UAG	0	(UCA)	UGG	16
											
Leu	CUU	56	Pro	CCU	60	His	CAU	32	Arg	CGU	6
(AAG)	CUC	22	(UGG)	CCC	33	(GUG)	CAC	34	(UCG)	CGC	5
	CUA	113		CCA	40	Gln	CAA	72		CGA	29
	CUG	17		CCG	10	(UUG)	CAG	6		CGG	5
											
Ile	AUU	237	Thr	ACU	76	Asn	AAU	80	Ser	AGU	7
(GAU)	AUC	109	(UGU)	ACC	79	(GUU)	AAC	101	(UCU)	AGC	18
											
Met	AUA	281		ACA	112	Lys	AAA	144		AGA	119
(CAU)	AUG	39		ACG	8	(UUU)	AAG	12		AGG	21
											
Val	GUU	32	Ala	GCU	73	Asp	GAU	28	Gly	GGU	27
(UAC)	GUC	15	(UGC)	GCC	33	(GUC)	GAC	30	(UCC)	GGC	19
	GUA	87		GCA	89	Glu	GAA	72		GGA	91
	GUG	21		GCG	3	(UUC)	GAG	8		GGG	34

^a^The number of codons used in 13 mitochondrial protein-coding genes

### Transfer RNA genes

**Figure 4:**
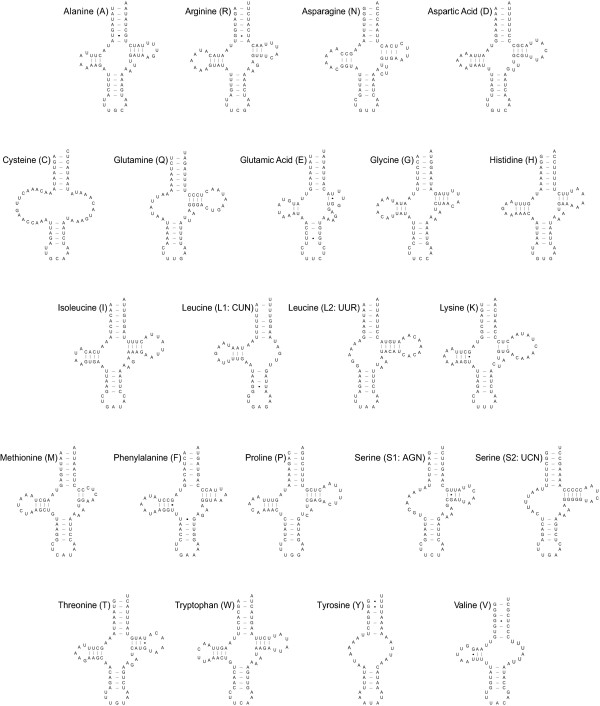
**Putative secondary structures of the 22 tRNAs identified in the mitochondrial genome of *Bugula neritina***. Bars indicate Watson-Click base pairings, and dots between G and U pairs mark canonical base pairings appearing in RNA.

The *B. neritina *mitochondrial genome contains 22 typical tRNA genes interspersed between the 2 rRNA and 13 protein-coding genes. This result differs from that of *F. hispidia*, which has only 21 tRNA genes because of the two serine tRNA genes, *trnS(agn) *and *trnS(ucn)*, *trnS(ucn) *is absent [31]. If we obtain more bryozoan mitochondrial genome data, it might be possible to provide reasonable evolutionary interpretations through further comparative analyses with respect to the absence/presence of *trnS(ucn)*. Thirteen of the 22 inferred *B. neritina *mitochondrial tRNAs have uniform features that are invariant in typical cloverleaf-shaped secondary structures with a 7-bp amino-acyl arm, 5-bp anticodon stem and 4-bp variable loop (Fig. 4). Two tRNAs [tRNA^Cys^, and tRNA^Tyr^] have no DHU arm or TψC arm. The TψC arm and variable loop are replaced by a single TV loop. In four tRNAs [tRNA^Gln^, tRNA^Leu(uur)^, tRNA^Ser(agn) ^and tRNA^Ser(ucn)^], the DHU arms are replaced by a loop. The unpaired DHU arm in tRNA^Ser(agn) ^has been considered a typical feature of animal mitochondrial genomes [25]. tRNA^Ser(ucn) ^with an unpaired DHU arm has also been reported for some protostomes: 2 nematodes (*Caenorhabditis elegans *and *Ascaris suum *[39]), 3 mollusks (1 chiton *K. tunicata *[40], 2 pulmonates *Cepaea nemoralis *and *Euhadra herklotsi *[41]), 2 brachiopods (*T. transversa *and *L. rubellus *[37,42]) and 1 annelid (*Lumbricus terrestris *[38]). We also found loss of the DHU arm from tRNA^Cys ^in the brachiopod *L. rubellus*, as in *B. neritina*.

Regardless of formation of a stable DHU arm, the first of 2 nts separating the amino-acyl stem from the DHU arm region is "T" in 14 tRNAs and the second is "A" in 19 tRNAs, and 1 nt separating the DHU arm region from the anticodon stem is "A" in 13 tRNAs. The 2 bp preceding the anticodon are always pyrimidines, with two exceptions – 'GU' in tRNA^Leu(cun) ^and 'AA' in tRNA^Tyr ^– and the 1 nt nearest the anticodon is "T" in 21 cases, the exception being 'A' in tRNA^Tyr^. The nt immediately after the anticodon is always a purine ["A" in 20 tRNAs] with two exceptions – tRNA^Glu ^and tRNA^Tyr ^have "U" in the same position. Among the 18 tRNAs that form a stable TψC arm, 4-nt variable arms typical of metazoan mitochondrial tRNAs were observed in 15 tRNAs, 5-nt variable arms in 2 tRNAs, tRNA^Asp ^and tRNA^Ser(agn)^, and 6-nt variable arms in tRNA^Glu^. The inferred anticodons for 20 tRNAs in *B. neritina *were the same as those in the other bryozoan, *F. hispida *(Fig. 4), but anomalies were detected in two tRNAs: tRNA^Tyr ^with AUA instead of GUA, and tRNA^Leu(cun) ^with GAG instead of UAG. The former has been reported for a few metazoans such as the predatory mite *Metaseiulus occidentalis *[43] and a onychophoran, *Epiperipatus biolleyi *[44], but the latter has never previously been reported for any metazoan. The tRNA^Leu(cun) ^with GAG may be considered an interesting feature unique to *B. neritina*. However, further experimental studies are needed to determine whether if it is a truly unique characteristic of *B. neritina*, or whether it results from a simple error in deducing the anticodon of tRNA^Leu(cun) ^from the nucleotide sequence of *trnL(cun)*.

### Ribosomal RNA genes

The two rRNA genes are generally separated by at least one gene (*trnV *in most of cases). In *B. neritina*, *rrnS *and *rrnL *are separated by *trnN *instead of *trnV*; *trnV *is located between *trnR *and *trnQ*. Assuming that the rRNA genes occupy all the available space between the adjacent genes, *rrnS *and *rrnL *are approximately 840 bp and 1,327 bp in length, respectively. The A+T contents of *rrnS *(69.1%) and *rrnL *(69.2%) are similar to the 70.0% of the whole mitochondrial genome. The total size (2,176 bp) of the *B. neritina *mitochondrial rRNAs was greater than those of the bryozoan *F. hispida *(1323 bp), 3 brachiopods (*T. transversa*, 1876 bp; *L. rubellus*, 1910 bp; *T. retusa*, 2057 bp), 2 annelids (*P. dumerilii*, 1962 bp; *L. terrestris*, 2030 bp) and a polyplacophoran mollusk *K. tunicata *(2101 bp), but less than those of a bivalve, *Mytilis edulis *(2189 bp), and a cephalopod, *L. bleekeri *(2312 bp).

### Phylogenetic position of bryozoans and lophophorate phylogeny

**Figure 5:**
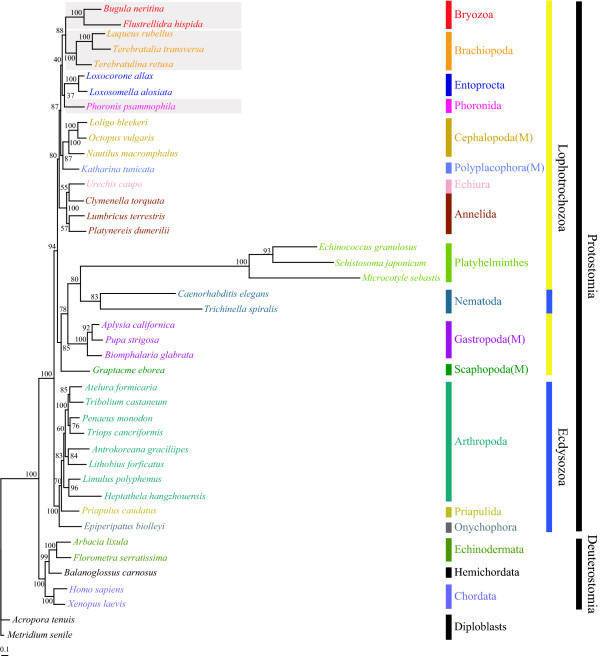
**Maximum likelihood tree inferred from amino acid sequences of 12 protein-coding genes of 42 metazoan mitochondrial genomes, showing weak support of the monophyly of lophophorates including Bryozoa, Brachiopoda, Phoronida and Entoprocta and a sister group relationship of Bryozoa and Brachiopoda**. The numbers above/below the branches indicate bootstrapping values (BP) that show node confidence values. Gray boxes indicate lophophorate members. *Metridium senile *and *Acropora tenuis *were used as outgroups. Refer to Table 5 for more detailed information and classification of the species used. "M" in parenthesis is an abbreviation of the phylum Molluska. The log likelihood value of the best tree is -66427.37.

As shown in Fig. 5 and [additional files 4, 5, 6], the first step of phylogenetic analysis (ML and BI) was performed on the basis of the nucleotide and amino acid sequences of 12 protein-coding genes in 42 metazoa (Table 5), in order to explore the phylogenetic position of bryozoans and lophophorate phylogeny within the Lophotrochozoa. All four trees showed that the two bryozoans (*B. neritina *and *F. hispida*) formed a strong monophyletic group (BP 100% in ML_aa _(Fig. 5) and ML_nt _[additional file 4], and BPP 1.0 in BI_aa _[additional file 5] and BI_nt _[additional file 6]). No tree supported lophophorate monophyly, except for the ML_aa _tree in Fig. 5, in which lophophorates including Entoprocta are grouped together with a weak node confidence value (BP 40%). The sister group of the bryozoan clade appeared to be brachiopods (BP 88 in ML_aa_, BP 48 in ML_nt _and BPP 0.86 in BI_nt_), except that the BI_aa _tree clustered Bryozoa with Phoronida [additional file 5]. As shown in Fig. 5 and [additional files 4, 5, 6], owing to possibly long-branch attraction artifacts (in particular, Nematoda and Platyhelminthes), all resultant ML and BI trees regardless of the data types employed showed unexpected groupings with extremely low node confidence values. In addition, phylogenetic trees inferred from nucleotide sequence data [additional files 4 and 6] had relatively lower node confidence values especially in deep branches. Amino acid-based trees (Fig. 5 and [additional file 5]) showed relatively higher node confidences in deep branches than the nucleotide-based trees [additional files 4 and 6].

**Table 5:** Species, classification and accession numbers used in the present phylogenetic analysis

**Taxon**	**Classification**	**Accession No.**
**Diploblasts**		
*Acropora tenuis*	Cnidaria, Anthozoa, Scleractinia	NC_003522
*Metridium senile*	Cnidaria, Anthozoa, Actiniaria	NC_000933
		
**Triploblasts**		
		
**Deuterostomes**		
*Arbacia lixula*	Echinodermata, Echinoidea	NC_001770
*Florometra serratissima*	Echinodermata, Crinoidea	NC_001878
*Balanoglossus carnosus*	Hemichordata, Enteropneusta	NC_001887
*Homo sapiens*	Chordata, Vertebrata, Primates	AC_000021
*Xenopus laevis*	Chordata, Vertebrata, Amphibia	NC_001573
		
**Protostomes**		
		
**Ecdysozoa**		
*Atelura formicaria*	Arthropoda, Hexapoda, Thysanura	NC_011197
*Tribolium castaneum*	Arthropoda, Hexapoda, Coleoptera	NC_003081
*Heptathela hangzhouensis*	Arthropoda, Chelicerata, Arachnida	NC_005924
*Limulus polyphemus*	Arthropoda, Chelicerata, Merostomata	NC_003057
*Lithobius forficatus*	Arthropoda, Myriapoda, Chilopoda	NC_002629
*Antrokoreana gracilipes*	Arthropoda, Myriapoda, Diplopoda	NC_010221
*Triops cancriformis*	Arthropoda, Crustacea, Notostraca	NC_004465
*Penaeus monodon*	Arthropoda, Crustacea, Decapoda	NC_002148
*Priapulus caudatus*	Priapulida, Priapulidae	NC_008557
*Epiperipatus biolleyi*	Onychopora, Peripatidae	NC_009082
*Caenorhabditis elegans*	Nematoda, Chromadorea	NC_001328
*Trichinella spiralis*	Nematoda, Enoplea	NC_002681
		
**Lophotrochozoa**		
*Bugula neritina*	Bryozoa, Gymnolaemata, Cheilostomata	AY690838(this study)
*Flustrellidra hispida*	Bryozoa, Gymnolaemata, Ctenostomata	NC_008192
*Terebratalia transversa*	Brachiopoda, Laqueidae	NC_003086
*Terebratulina retusa*	Brachiopoda, Cancellothyrididae	NC_000941
*Laqueus rubellus*	Brachiopoda, Laqueidae	NC_002507
*Phoronis psammophila*	Phoronida, Phoroniformea	AY368231(partial)
*Loxocorone allax*	Entoprocta, Loxosomatidae, Loxocorone	NC_010431
*Loxosomella aloxiata*	Entoprocta, Loxosomatidae, Loxosomella	NC_010432
*Aplysia californica*	Molluska, Gastropoda, Opisthobranchia	NC_005827
*Biomphalaria glabrata*	Molluska, Gastropoda, Pulmonata	NC_005439
*Pupa strigosa*	Molluska, Gastropoda, Opisthobranchia	NC_002176
*Graptacme eborea*	Molluska, Scaphopoda, Dentaliida	NC_006162
*Loligo bleekeri*	Molluska, Cephalopoda, Coleoidea	NC_006321
*Nautilus macromphalus*	Molluska, Cephalopoda, Nautiloidea	NC_007980
*Octopus vulgaris*	Molluska, Cephalopoda, Coleoidea	NC_006353
*Katharina tunicate*	Molluska, Polyplacophora	NC_001636
*Clymenella torquata*	Annelida, Polychaeta, Capitellida	NC_002322
*Lumbricus terrestris*	Annelida, Clitellata, Haplotaxida	NC_001673
*Platynereis dumerilii*	Annelida, Polychaeta, Phyllodocida	NC_000931
*Microcotyle sebastis*	Platyhelminthes, Trematoda, Monogenea	NC_009055
*Schistosoma japonicum*	Platyhelminthes, Trematoda, Digenea	NC_002544
*Echinococcus granulosus*	Platyhelminthes, Cestoda, Eucestoda	NC_008075
*Urechis caupo*	Echiura, Xenopneusta, Urechidae	NC_006379

**Figure 6:**
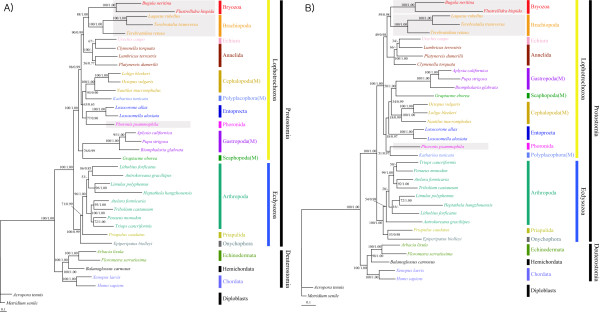
**Maximum likelihood trees inferred from amino acid (A) or nucleotide (B) sequences of 12 protein-coding genes in 37 metazoan mitochondrial genomes, showing a monoclade of Bryozoa and Brachipoda, a sister group relationship of Bryozoa + Brachiopoda and Annelida-Echiura, non-monophyly of lophophorates, and a close relationship of Phoronida and Entoprocta (or *Katharina tunicate*)**. The numbers above and below the branches indicate bootstrapping values in percentage (BP) and Bayesian posterior probabilities (BPP) in order, which show node confidence values. Because the BI tree was very similar to the ML tree, only the ML tree is presented here and the BPP values of the BI tree are shown with BP values of the ML tree on each node. Gray boxes indicate lophophorate members. *Metridium senile *and *Acropora tenuis *were used as outgroups. Refer to Table 5 for more detailed information and classification of the species used. M in parenthesis is an abbreviation of the phylum Molluska. The log likelihood values of the best trees are -72906.37 in (A) and -106791.00 in (B).

To resolve the problem of long-branch attraction, 2 nematodes and 3 platyhelminths were excluded from the first data set for the second-round phylogenetic analyses. The ML and BI trees newly obtained with the reduced data set, including 37 taxa comprising 35 protostomes (20 lophotrochzoans and 10 ecdysozoans), 5 deuterostomes and 2 primitive metazoans (outgroup taxa) were improved, robust and reliable with higher nodal support values. Within the Lophotrochozoa, all four trees (Fig. 6) showed that the monophylies of the two bryozoans (*B. neritina *and *F. hispida*) and the three brachiopods (*T. transversa*, *L. rubellus*, *T. retusa*) were strongly supported with strong nodal supports (BP 100% in ML_aa _and ML_nt _and BPP 1.0 in BI_aa _and BI_nt_). In all four trees shown in Fig. 6, the strong monophyletic bryozoan clade, within the Lophotrochozoa, was grouped with a monophyletic brachiopod clade (BP 88% and 59% in ML_aa _and ML_nt _and BPP 1.0 and 0.98 in BI_aa _and BI_nt_, respectively). The clade of Bryozoa + Brachiopoda was grouped with the clade of Annelida including Echiura as a subtaxon (BP 90% and 49% in ML_aa _and ML_nt _and BPP 0.99 and 0.98 in BI_aa _and BI_nt_, respectively). *P. psamophila *(Phoronida) was clustered with Entoprocta in ML_aa _(BP 77%) and BI_aa_(BPP 0.90), which is consistent with the result of Yokobori et al. [45] based on mitochondrial protein-coding genes. In contrast, *P. psamophila *was grouped with a chiton, *K. tunicate*, in ML_aa _(BP 51%) and BI_aa _(BPP 0.97). This indicates that the phylogenetic positions of Phoronida, Entoprocta and *K. tunicata *are still ambiguous. No tree in Fig. 6 supports lophophorate monophyly.

The results of the present phylogenetic analyses revealed that lophophorates are placed with mollusks and annelids as members of a monophyletic lophotrochozoan group. This is consistent with evidence from 18S rRNA [15,17,46], Hox genes [20], Na/K ATPase α-subunit [47] and molecular data [14-18,29]. Therefore, it strongly suggests that the long-held view inferred from morphological data [10] that deuterostomes have affinity with Bryozoa and the other two lophophorates should be refuted. Recent reports on lophophorate phylogeny based on SSU rRNA gene sequences [24,48] coincide with the present result in that lophophorates are unambiguously affiliated with protostomes rather than deuterostomes.

Contrary to the present findings, which cluster Bryozoa with Brachiopoda, some previous SSU rRNA-based results have shown that brachiopods and phoronids (called the subphylum 'Phoroniformea', 'Brachiozoa' or 'Poronozoa') form a separate clade from the bryozoans and even suggest that phoronids may be members of the inarticulate brachiopods [11,15,17,19,21,23,30,48,49]. However, the present trees did not show the Brachiozoa grouping at all.

To clarify the statistical support for each grouping such as the monophylies of Brachiozoa, Lophophorata, the old-concept Bryozoa (comprising Entoprocta and Ectoprocta) [50,51] and the sister group Bryozoa + Brachiopoda, we performed tree topology tests (Table 6). The results indicate that on the basis of statistical probability, the sister group of Bryozoa + Brachiopoda could be the Annelida-Echiura or the Phoronida + Entoprocta clade. If the latter is a true phylogeny, lophophorate monophyly including Entoprocta may be supported. The tree topology test is likely to indicate that Brachiozoa (= Brachiopoda + Phoronida) and the recently reinstated old-concept Bryozoa may be refuted, but according to the present data the sister group of Bryozoa is Brachiopoda (Table 6).

**Table 6:** Topology test results

**Hypothesis**	**Phylogenetics Hypothesis**	**ELW Test**
Monophyly of Bryozoa and Brachiopoda	**(((Br, Bc),(An-Ec)),(En, Ph))**	0.4837*

//	**(((Br, Bc),(En, Ph)),(An-Ec))**	0.4939*

//	((((Br, Bc), Ph),(An-Ec)), En)	0.0135

//	((((Br, Bc), Ph), En),(An-Ec))	0.0135

Old-concept Bryozoa	((En, Br), Bc,(An-Ec), Ph)	0.0160

Brachiozoa	(((Bc, Ph), Br, En),(An-En))	0.0005
	
	((Bc, Ph), Br,(An-Ec), En)	0.0002

Asterisks (*) mark values for the topologies included in the 0.95 confidence set (ELW of the tree topologies with the highest confidence levels that added up to 0.95). The two bold-letter lines are accepted and the others are refuted.

Br, Bryozoa (*Bugula*+*Flustrellidra*); En, Entoprocta (*Loxocorone*+*Loxosomella*); Bc, Brachiopoda [((*Laqueus*, *Terebratalia*), *Terebratulina*)]; Ph, Phoronida (*Phoronis*); An, Annelida (*Clymenella*,(*Lumbricus*, *Platynereis*)); Ec, Echiura (*Urechis*).

Despite intensive phylogenetic analyses, phylogenetic relationships among lophotrochozoan members including lophophorates and others unfortunately remain unclear because there are conflicts among the phylogenetic trees reconstructed by different tree-making methods, with different data types and with different taxon samplings (Figs. 5 and 6 and [additional files 4, 5, 6]). The phylogeny signal of mitochondrial genome nucleotides and/or amino acids alone may be unable to resolve what may have been a relatively rapid radiation during the Cambrian [52,53]. Recently, to overcome such limitations, huge EST data sets from a number of metazoans have been employed to resolve metazoan phylogeny [49]. The results still left the phylogenetic position of bryozoans unclear, and lophophorates did not form a monophyletic group. Further more intensive studies seem to be necessary to resolve the exact phylogenetic position of the bryozoans and to examine the question of lophophorate monophyly.

## Conclusion

This study presents the complete mitochondrial genome of a cheliostome bryozoan, *B. neritina*. Comparison of the orders of the protein-coding genes showed the possibility that three lophophorates are closely related, including *K. tunicata*. The present phylogenetic analyses suggest the probable relationships ((Bryozoa, Brachiopoda), Annelida-Echiura), or ((Bryozoa, Brachiopoda), (Phoronida, Entoprocta)), but the phylogenetic position of phoronids is still ambiguous. Consequently, the results seem to imply that the three lophophorate members did not form a monophyletic group in the phylogenetic trees and this possibility was also refuted statistically. However, according to the tree topology test, lophophorate monophyly including Entoprocta – ((Bryozoa, Brachiopoda), (Phoronida, Entoprocta)) – was not refuted. In addition, Brachiozoa (= Brachiopoda + Phoronida) and the recently-reinstated old-concept Bryozoa may be refuted, but according to the present data the sister group of Bryozoa is Brachiopoda (Table 6). However, because only a few samples of lophophorates were used here and there were some conflicts among the resultant trees, it is better to postpone a final decision on the phylogenetic position of bryozoans and on lophophorate phylogeny. Until more mitochondrial genomes become available and until we know more about the evolution of these organelle genomes, we may not come to any conclusion with respect to the monophyly or polyphyly of the lophophorates.

## Methods

### Specimen collection and DNA extraction

*Bugula neritina *(Bryozoa) was collected at Cheonsuman, Taean Gun, Chungnam Province, Korea. Total genomic DNA was extracted using a DNeasy tissue kit (QIAGEN Co., Hilden, Germany) following the manufacturer's protocol.

### PCR amplification and cloning

The entire *Bugula *mitochondrial genome was amplified by two kinds of overlapping polymerase chain reactions (PCR). The PCR strategy was as follows: the ca. 2.5 kb fragment from *cox1 *to *rrnL *was amplified with previously reported universal primers, 16SA (5'-CGC CTG TTT ATC AAA AAC AT-3'; [54]) and HCO2198 (5'-TAA ACT TCA GGG TGA CCA AA AAA -3'; [55]). From the newly-sequenced ca. 2.5-kb sequences, the following two *Bugula*-specific primers were designed to amplify the remaining part (ca. 13.5 kb) of the mitochondrial genome: bnCOI (5'-AGC CAT TTT CTC TTT ACA CCT TGC-3') and bn16S (5'-TCA CTA CAA ACT CTA CAG GGT CTT-3').

The 2.5-kb PCR product was directly ligated to the pGEM T-easy vector (Promega), and the 13.5-kb PCR product was digested with *Pst*I, generating four fragments (approximately 0.9, 2.7, 2.7 and 7 kb). The two internal *Pst*I-restricted fragments (0.9 kb and 2.7 kb) were ligated into *Pst*I-digested pUC19 vector and both the end fragments (2.7 kb and 7 kb) with A-tailings were ligated into the modified, *Pst*I-digested pGEM T-easy vector (Promega Co.). All ligates were cloned with *Escherichia coli *DH5α strain. Correct recombinants were selected by the blue/white colony selection method using X-gal and IPTG. Plasmid DNAs were purified using an AtmanBio Plasmid Miniprep Kit (Takara Co., Japan).

### Sequencing and sequence analysis

The purified plasmid DNA was sequenced using a primer walking method with the ABI PRISM BigDye terminator system and analyzed on an ABI3700 model automatic sequencer (Genotech Co., Korea). DNA sequences were analyzed using GeneJockey II, Version 1.6 (BIOSOFT Inc., Cambridge, UK). Thirteen mitochondrial protein-coding genes were initially identified by a BLAST comparison with other animal mitochondrial genomes, with start codons inferred as eligible in-frame start codons corresponding at least to the extent of alignment that does not overlap the upstream gene. Protein gene termini were inferred to be at the first in-frame stop codon unless this was located within the sequence of a downstream gene. Otherwise, a truncated stop codon (T or TA) adjacent to the beginning of the downstream gene was designated the termination codon, assuming that it could be completed by polyadenylation after transcript cleavage [56]. Ribosomal RNAs were identified by a BLAST search. A preliminary screening for tRNA genes was carried out using tRNAscan-SE, version 1.1 [57]. The tRNA genes that were not identified in this way were visually identified by inspection of anticodon sequences and their proposed cloverleaf secondary structures [58]. The sequence data obtained here are available from DDBJ/EMBL/GenBank under accession number AY690838.

### Phylogenetic analysis

For the first step in the present phylogenetic analyses, we employed 40 protostomes and deuterostomes as ingroup taxa and 2 primitive metazoans as outgroup taxa, as listed in Table 5. When we selected the taxa for the present analyses, we tried to include all the lophotrochzoans for which complete mitochondrial genomes had already been sequenced. Some representative and/or slowly-evolving ecdysozoans and deuterostomes were included as reference taxa. All mitochondrial genome sequences obtained from members of the phyla Bryozoa (2 species), Brachiopoda (3), Phoronida (1), Echiura (1) and Entoprocta (2) were used here. However, since complete mitochondrial genome sequences from a number of members of the phyla Molluska (45), Platyhelminthes (28), and Annelida (8) have been determined, we selected only 3 each from Annelida and Platyhelminthes and 8 from Molluska, in order to reduce the calculation time in the present analyses. Those selected are representative and/or slowly-evolving ones in each phylum. *Paraspadella gotoi *and *Spadella cephaloptera *(Phyum Chaetognatha) and *Cephalothrix rufifrons *(Phylum Nemertea) were not included in the present analyses because they do not have *atp6 *and *atp8*, or have some genes that are as yet unidentified.

The nucleotide and amino acid sequences of the 12 protein-coding genes were used for the analyses. Only the 12 multiple alignment subsets of these sequences were created using a Clustal X multiple alignment program [59] under the default option. Only well-aligned, conserved alignment sites were extracted from each alignment subset using the Gblock program [60] with the default option. The conserved blocks extracted were subsequently concatenated into a single, unified, large alignment set with the Gblock program. In the second-round phylogenetic analyses, to resolve the problem of long-branch attraction, 5 taxa (2 nematodes and 3 platyhelminths) showing extremely long branches (Fig. 5 and [additional file 4]) were excluded from the original data set used in the first step. In total, the nucleotide and amino acid sequences of the mitochondrial protein-coding genes for 37 taxa were aligned and conserved blocks were extracted as described above.

For the first-round phylogenetic analyses with 42 metazoan mitochondrial genomes, the refined alignments (1735 aa and 4470 nt positions in length) were subjected to two different tree-making algorithms: the maximum likelihood (ML) and Bayesian inference (BI) methods. For phylogenetic analyses based on amino acid sequences, rather than using hierarchical likelihood ratio tests to select the best-fitting model for the evolution of sequences, and to calculate the related parameter values (I and Ã), ProtTest ver. 1.3 was used under the Akaike Information Criterion (AIC) because it has several important advantages [61]. Among the 36 models implemented in this program, the best-fitting model selected was MtArt [62] with among-site substitution-rate heterogeneity described by a gamma distribution (Ã = 0.732) and a fraction of sites constrained to be invariable (I= 0.072). For phylogenetic analyses based on nucleotide sequences, the best-fitting evolutionary model was estimated by Model Test 3.6 [63], from which the GTR+G+I (general time reversible model + among site rate variation + invariable sites) model was selected. Model Test 3.6 was also used to estimate the substitution rate parameters between nucleotides (AC 1.64479, AG 3.36847, AT 1.24161, CG 3.28174, CT 3.48682, and GT 1.00000) for the GTR model, base frequencies (A = 0.244605, C = 0.141275, G = 0.184743, T = 0.429377), assumed proportion of invariable sites (I = 0.126031), and the shape parameter (alpha) of the among-site rate variation (G = 0.665080).

For the second-round phylogenetic analyses with 35 protostomes and deuterostomes and 2 outgroup taxa, the refined alignments (2127 aa and 4965 nt positions in length) were subjected to the two different tree-making algorithms, ML and BI. For phylogenetic analyses based on amino acid sequences, MtArt was selected as the best-fitting model [62] with among-site substitution-rate heterogeneity described by a gamma distribution (Γ = 0.714) and a fraction of sites constrained to be invariable (I = 0.1511). For phylogenetic analyses based on nucleotide sequences, GTR+G+I (general time reversible model + among site rate variation + invariable sites) was selected as the best-fitting model. The substitution rate parameters between nucleotides were AC 1.08325, AG 3.02089, AT 1.20831, CG 2.51010, CT 2.92091, and GT 1.00000 for the GTR model, the base frequencies were A = 0.259281, C = 0.176486, G = 0.176848, T = 0.387385, the invariable site parameter (I) was 0.105884, and the shape parameter (alpha) of the among-site rate variation was G = 0.593221.

All the parameters estimated were then employed for ML and BI analyses in the first and second round phylogenetic analyses, respectively. Four rate categories were used in the present study. The ML analysis was carried out using PHYML v2.4.4 [64] and Treefinder [65]. The bootstrap proportions in percentage (BP) of the ML tree were obtained with 500 replicates by the fast-ML method using PHYML and Treefinder. The BI analysis was carried out using the MrBayes v3.0b4 program [66] with the following options: 1,000,000 generations, 4 chains (1 hot and 3 cold) and a burn-in step of the first 10,000. The node confidence values of the BI tree were presented with Bayesian posterior probabilities (BPP).

Statistical confidence values for possible groupings of the ML tree based on the amino acid residues of 12 protein-coding genes were computed by applying expected likelihood weights (ELWs) [67] to all local rearrangements (LR) of tree topology around an edge (1,000 replicates) using the program TREEFINDER.

## Abbreviations

*atp6 *and *atp8*: genes for the ATPase subunits 6 and 8; *cox1-cox3*: genes for cytochrome C oxidase subunits I-III; *cob*: a gene for apocytochrome b; *nad1-nad6 *and *nad4L*: genes for NADH dehydrogenase subunits 1–6 and 4L; *rrnS *and *rrnL*: genes for 12S and 16S rRNAs; *trnX*: where X is replaced by single-letter amino acid abbreviations of the corresponding amino acids; *trnL1 *and *trnL2*: genes for tRNA^Leu(UUR) ^(anticodon TAA) and tRNA^Leu(CUN) ^(anticodon TAG): respectively; *trnS1 *and *trnS2*: genes for the tRNA^Ser(UCN) ^(anticodon TGA) and tRNA^Ser(AGN) ^(anticodon GCT): respectively; ML: the maximum likelihood method; BI: Bayesian inference; BPP: Bayesian posterior probabilities; BP: bootstrap proportions; ML_nt_: the maximum likelihood tree inferred from nucleotide sequences; ML_aa_: the maximum likelihood tree inferred from amino acid sequences; BI_nt_: the Bayesian inference tree inferred from nucleotide sequences; BI_aa_: the Bayesian inference tree inferred from amino acid sequences.

## Competing interests

The authors declare that they have no competing interests.

## Authors' contributions

KHJ and UWH made substantial contributions to the conception and design of the study, acquisition of the data, and analysis and interpretation of the data. KHJ wrote the early draft of this manuscript, and UWH revised and rewrote all parts of the manuscript. Both authors read and approved the final version of the manuscript. UWH gave final approval of the version to be published.

## Supplementary Material

Additional file 1 **A hairpin-loop structure of a noncoding region NC1 in the mitochondrial genome of a bryozoan, *****Bugula neritina.*** Click here for file

Additional file 2 **AT-skew of mitochondrial protein-coding and ribosomal RNA genes of 14 lophotrochozoan species.** Click here for file

Additional file 3 **CG-skew of mitochondrial protein-coding and ribosomal RNA genes of 14 lophotrochozoan species.** Click here for file

Additional file 4 **Maximum likelihood tree inferred from nucleotide sequences of 12 protein-coding genes of 42 metazoan mitochondrial genomes, showing non-monophyly of lophophorates and a sister group relationship of Bryozoa and Brachiopoda**. The numbers above/below the branches indicate bootstrapping values (BP) that show node confidence values. Gray boxes indicate lophophorate members. *Metridium senile *and *Acropora tenuis *were used as outgroups. Refer to Table 5 for more detailed information and classification of the species used. M in a parenthesis is an abbreviation of the phylum Molluska. The log likelihood value of the best tree is -112314.88. Click here for file

Additional file 5 **Bayesian Inference tree inferred from amino acid residues of 12 protein-coding genes of 42 metazoan mitochondrial genomes**. The numbers above/below the branches indicate Bayesian posterior probabilities (BPP) that show node confidence values. *Metridium senile *and *Acropora tenuis *were used as outgroups. The log likelihood value of the best tree is -68516.902. Refer to Table 5 for more detailed information. Click here for file

Additional file 6 **Bayesian Inference tree inferred from nucleotide sequences of 12 protein-coding genes of 42 metazoan mitochondrial genomes**. The numbers above/below the branches indicate Bayesian posterior probabilities (BPP) that show node confidence values. *Metridium senile *and *Acropora tenuis *were used as outgroups. The log likelihood value of the best tree is -112068.205. Refer to Table 5 for more detailed information. Click here for file
