# Supplementary material for: Complete mitochondrial genome of Bugula neritina (Bryozoa, Gymnolaemata, Cheilostomata): phylogenetic position of Bryozoa and phylogeny of lophophorates within the Lophotrochozoa
Source: BMC Genomics. 2009 Apr 21;10:167. doi: 10.1186/1471-2164-10-167 (PMC2678162; doi:10.1186/1471-2164-10-167)
Supplement: Additional file 2 — AT-skew of mitochondrial protein-coding and ribosomal RNA genes of 14 lophotrochozoan species. [file 1471-2164-10-167-S2.docx]

|  | **atp6** | **atp8** | **cox1** | **cox2** | **cox3** | **cob** | **nad1** | **nad2** | **nad3** | **nad4** | **nad4L** | **nad5** | **nad6** | **rrnL** | **rrnS** |
| --- | --- | --- | --- | --- | --- | --- | --- | --- | --- | --- | --- | --- | --- | --- | --- |
| *Bugula neritina* | -0.054 | -0.052 | -0.079 | 0.095 | 0.063 | 0.004 | 0.083 | 0.040 | -0.073 | 0.076 | 0.013 | 0.121 | -0.055 | 0.203 | 0.216 |
| *Flustrellidra hispida* | -0.120 | -0.161 | -0.112 | -0.190 | -0.142 | -0.128 | -0.115 | -0.107 | -0.192 | -0.184 | -0.176 | -0.044 | -0.158 | 0.054 | 0.046 |
| *Terebratalia transversa* | **-0.466** | -0.247 | **-0.363** | **-0.320** | **-0.412** | **-0.359** | **-0.396** | **-0.364** | **-0.450** | **-0.464** | **-0.566** | **-0.447** | **-0.448** | -0.084 | -0.064 |
| *Terebratulina retusa* | -0.084 | 0.084 | 0.009 | 0.118 | -0.057 | -0.103 | -0.026 | -0.071 | -0.150 | -0.077 | -0.139 | 0.025 | -0.068 | 0.270 | 0.291 |
| *Laqueus rubellus* | **-0.397** | -0.163 | **-0.325** | **-0.365** | **-0.399** | **-0.362** | **-0.355** | **-0.413** | **-0.520** | **-0.368** | **-0.365** | **-0.364** | **-0.398** | 0.010 | -0.036 |
| *Phoronis psammophila* | -0.211 | -0.189 | -0.163 | -0.149 | -0.276 | -0.244 | -0.207 | -0.232 | **-0.324** | -0.190 | -0.235 | -0.129 | -0.229 | -0.049 | -0.073 |
| *Platynereis dumerilii* | -0.089 | 0.028 | -0.091 | 0.035 | -0.157 | -0.083 | -0.007 | 0.011 | -0.104 | -0.030 | 0.005 | -0.060 | -0.079 | 0.069 | 0.122 |
| *Lumbricus terrestris* | -0.065 | -0.083 | -0.119 | 0.038 | -0.173 | -0.099 | -0.085 | -0.029 | -0.127 | -0.046 | -0.125 | -0.083 | -0.113 | 0.137 | 0.175 |
| *Clymenella torquata* | -0.056 | -0.035 | -0.068 | 0.057 | -0.043 | -0.086 | -0.087 | -0.030 | -0.131 | -0.075 | -0.101 | -0.050 | -0.047 | 0.157 | 0.106 |
| *Urechis caupo* | -0.029 | -0.009 | -0.046 | -0.077 | -0.037 | -0.059 | -0.045 | -0.092 | -0.041 | 0.012 | -0.124 | 0.012 | -0.109 | 0.203 | 0.194 |
| *Aplysia californica* | -0.152 | -0.009 | -0.256 | -0.077 | -0.159 | -0.273 | **-0.327** | -0.291 | -0.251 | -0.250 | -0.283 | -0.220 | **-0.313** | -0.053 | 0.221 |
| *Biomphalaria glabrata* | -0.127 | -0.119 | -0.115 | -0.076 | -0.155 | -0.223 | -0.213 | -0.195 | -0.152 | -0.194 | -0.202 | -0.201 | -0.249 | -0.028 | 0.006 |
| *Pupa strigosa* | **-0.347** | 0.080 | -0.207 | -0.115 | -0.233 | -0.177 | -0.171 | -0.222 | -0.285 | -0.156 | -0.267 | -0.203 | -0.224 | 0.004 | 0.075 |
| *Katharina tunicata* | -0.123 | **-0.363** | -0.279 | -0.212 | **-0.312** | -0.131 | -0.213 | **-0.343** | -0.152 | -0.194 | -0.019 | 0.009 | -0.175 | 0.083 | 0.064 |

**Additional file 2: AT skew of mitochondrial protein-coding and ribosomal RNA genes of 16 lophotrochozoan species**

Bold letters mark the values of the genes over 0.300.
