# Supplementary material for: Complete mitochondrial genome of Bugula neritina (Bryozoa, Gymnolaemata, Cheilostomata): phylogenetic position of Bryozoa and phylogeny of lophophorates within the Lophotrochozoa
Source: BMC Genomics. 2009 Apr 21;10:167. doi: 10.1186/1471-2164-10-167 (PMC2678162; doi:10.1186/1471-2164-10-167)
Supplement: Additional file 3 — CG-skew of mitochondrial protein-coding and ribosomal RNA genes of 14 lophotrochozoan species. [file 1471-2164-10-167-S3.docx]

**Additional file 3: CG skew of mitochondrial protein-coding and ribosomal RNA genes of 16 lophotrochozoan species**

|  | **atp6** | **atp8** | **cox1** | **cox2** | **cox3** | **cob** | **nad1** | **nad2** | **nad3** | **nad4** | **nad4L** | **nad5** | **nad6** | **rrnL** | **rrnS** |
| --- | --- | --- | --- | --- | --- | --- | --- | --- | --- | --- | --- | --- | --- | --- | --- |
| *Bugula neritina* | 0.144 | **0.517** | 0.022 | 0.122 | 0.106 | 0.194 | 0.242 | 0.281 | 0.111 | 0.244 | 0.178 | 0.285 | 0.220 | 0.032 | 0.062 |
| *Flustrellidra hispida* | 0.266 | 0.172 | 0.112 | **-0.320** | 0.099 | 0.142 | 0.122 | 0.033 | 0.087 | 0.167 | 0.059 | 0.193 | 0.194 | -0.182 | -0.036 |
| *Terebratalia transversa* | **-0.378** | **-0.410** | -0.228 | -0.279 | -0.276 | -0.244 | **-0.380** | **-0.503** | **-0.448** | **-0.402** | **-0.570** | **-0.471** | **-0.538** | -0.235 | -0.241 |
| *Terebratulina retusa* | **0.451** | **0.397** | 0.256 | **0.317** | 0.259 | **0.321** | **0.379** | **0.452** | **0.361** | **0.329** | 0.252 | **0.402** | 0.270 | 0.158 | 0.204 |
| *Laqueus rubellus* | **-0.329** | **-0.586** | -0.170 | -0.259 | **-0.353** | -0.203 | **-0.326** | **-0.436** | **-0.348** | -0.187 | **-0.383** | **-0.348** | **-0.465** | -0.222 | -0.150 |
| *Phoronis psammophila* | -0.077 | 0.200 | -0.049 | -0.087 | -0.043 | 0.003 | 0.006 | -0.078 | -0.053 | -0.023 | 0.000 | -0.046 | -0.038 | 0.188 | 0.116 |
| *Platynereis dumerilii* | 0.160 | **0.472** | 0.078 | 0.109 | 0.052 | 0.137 | 0.200 | 0.209 | 0.221 | 0.178 | 0.072 | 0.187 | 0.281 | 0.124 | 0.168 |
| *Lumbricus terrestris* | **0.379** | **0.529** | 0.135 | 0.142 | 0.152 | 0.199 | 0.141 | 0.242 | 0.165 | 0.264 | 0.162 | 0.222 | **0.477** | 0.115 | 0.073 |
| *Clymenella torquata* | 0.262 | **0.556** | 0.091 | 0.177 | 0.078 | 0.224 | 0.173 | 0.247 | **0.333** | **0.320** | 0.238 | 0.293 | **0.365** | 0.136 | 0.077 |
| *Urechis caupo* | **0.347** | **0.438** | 0.160 | 0.237 | 0.195 | 0.250 | 0.200 | **0.343** | 0.206 | **0.354** | 0.260 | **0.324** | **0.365** | 0.180 | 0.158 |
| *Aplysia californica* | -0.014 | 0.000 | -0.095 | -0.109 | -0.028 | -0.022 | -0.190 | -0.159 | 0.000 | -0.105 | -0.065 | -0.143 | -0.176 | -0.177 | -0.186 |
| *Biomphalaria glabrata* | 0.145 | 0.273 | -0.115 | -0.059 | -0.005 | -0.066 | -0.162 | -0.228 | 0.046 | -0.135 | -0.091 | -0.182 | **-0.303** | -0.162 | -0.006 |
| *Pupa strigosa* | 0.000 | **0.317** | -0.086 | -0.062 | -0.074 | 0.020 | -0.113 | -0.183 | -0.113 | -0.118 | -0.088 | -0.066 | -0.209 | -0.010 | -0.033 |
| *Katharina tunicata* | **-0.340** | **-0.375** | **-0.304** | -0.295 | -0.218 | 0.183 | 0.157 | **-0.409** | **-0.351** | 0.245 | 0.134 | 0.253 | 0.191 | -0.015 | 0.022 |

Bold letters mark the values of the genes over 0.300.
